# Predictive Factors of Functional Prognosis in Patients with Rhegmatogenous Retinal Detachment Treated by Pars Plana Vitrectomy—A Retrospective Study

**DOI:** 10.3390/diagnostics16111696

**Published:** 2026-05-30

**Authors:** George Chereji, Vlad-Florin Chelaru, Simona Delia Nicoară

**Affiliations:** 1 Doctoral School of Medicine, “Iuliu Hatieganu” University of Medicine and Pharmacy, 8, V. Babes, Str. 43, 400012 Cluj-Napoca, Romania; 2 Department of Neurosciences, “Iuliu Hatieganu” University of Medicine and Pharmacy, 8, V. babes, Str. 43, 400012 Cluj-Napoca, Romania; 3 RoNeuro Institute for Neurological Research and Diagnostic, Mircea Eliade Str. 37, 400354 Cluj-Napoca, Romania; 4 Department of Ophthalmology, “Iuliu Hatieganu” University of Medicine and Pharmacy, 8, V. Babes, Str. 43, 400012 Cluj-Napoca, Romania; 5 Ophthalmology Clinic, Emergency County Hospital, 3–5 Clinicilor Str., 400006 Cluj-Napoca, Romania

**Keywords:** rhegmatogenous retinal detachment, pars plana vitrectomy, predictive factors, visual and functional outcome

## Abstract

**Background/Objectives**: Rhegmatogenous retinal detachment (RRD) is a surgical emergency that causes vision loss if not properly treated. Pars plana vitrectomy (PPV) is the preferred procedure for most complex RRD cases, with a high success rate. This study aimed to evaluate the significance of predictive factors and their impact on postoperative best corrected visual acuity (BCVA) in patients undergoing PPV for RRD. **Methods**: In this retrospective study, 136 eyes from 135 patients who underwent PPV for RRD were examined. A robust fitting algorithm for linear models was used to assess the influence of preoperative patient-related factors on the functional outcomes of PPV for RRD. **Results**: Various factors were analyzed, ranging from demographic parameters, such as gender and residency, to more complex ocular findings, including BCVA, duration of symptoms, description of the RRD, retinal tears, macular status, presence of proliferative vitreoretinopathy (PVR), epiretinal membrane, or macular hole. **Conclusions:** Duration of symptoms, phacoemulsification during follow-up, and preoperative BCVA showed a statistically significant correlation with postoperative BCVA. Lens status, macular status, extent of retinal detachment, number of tears, and PVR did not influence the postoperative outcome.

## 1. Introduction

Rhegmatogenous retinal detachment (RRD) is a surgical emergency that causes vision loss if not treated properly. Usually, complex surgery is needed to restore the retina to its normal position. The average annual incidence of RRD in Europe is estimated at 14.52 cases per 100,000 people [1]. A 3-year retrospective study conducted in a developing country showed that RRD surgery was the most common procedure performed in a vitreoretinal operating theater, accounting for 45.1% of all PPVs which cost the limited human and financial resources of the country [2].

RRD is defined as a separation between the neurosensitive retina and the retinal pigment epithelium (RPE) caused by one or more retinal tears/breaks. In time, in various pathological conditions such as high myopia or vitreous hemorrhage following surgeries like cataract extraction, or simply due to the aging and degeneration of the vitreous (syneresis), the hyaluronic acid content of the vitreous gel decreases, leading to changes in the vitreous anatomy and vitreoretinal adhesion. Persistent vitreoretinal adhesion can cause traction and the formation of retinal breaks which separate the neuroretina from the underlying RPE due to fluid influx [3,4].

The main surgical methods for managing RRD are pneumatic retinopexy, scleral buckle (SB), and pars plana vitrectomy (PPV). PPV is regarded as the preferred procedure for treating RRD, especially in pseudophakic eyes or complex retinal detachment, with success rates ranging from 70 to 90% in uncomplicated cases [5,6] and up to 97% in overall success. PPV provides better visibility of the retinal periphery and facilitates identifying breaks, leading to quicker foveal reattachment [7,8]. However, despite this procedure, visual recovery often remains less satisfactory.

Many studies have identified several prognostic factors linked to visual recovery after RRD surgery. These include duration of symptoms [9,10], preoperative best corrected visual acuity (BCVA) [11,12,13], extent of retinal detachment [14,15], macular status [16], macular hole [17], and proliferative vitreoretinopathy (PVR) [18]. These factors are associated with variable final functional outcomes.

The main hypothesis of our study was to outline that functional recovery after RRD surgery is determined by a combination of modifiable and non-modifiable prognostic factors, with macular involvement, symptom duration, and baseline BCVA being the strongest predictors for visual outcome. Our retrospective study aimed to evaluate the significance of predictive factors and their impact on postoperative BCVA in patients undergoing vitrectomy for RRD. Understanding these variables is essential for patient counseling before surgery, especially considering patients’ concerns about their potential visual prognosis.

## 2. Methods

### 2.1. Patient Selection

A retrospective review of records from patients operated on for RRD was conducted between January 2021 and December 2024 at the Emergency County Hospital of Cluj-Napoca, Department of Ophthalmology. This study was performed with the approval of the Ethics Committees belonging to the “Iuliu Hatieganu” University of Medicine and Pharmacy of Cluj Napoca (No.176 from 24 July 2025) and the Emergency County Hospital of Cluj-Napoca (No.11281 from 30 March 2026), and the research was conducted in accordance with the Declaration of Helsinki. Written informed consent was obtained from all patients before the surgical procedure, as well as for collecting data.

Inclusion criteria required a diagnosis of primary RRD in patients aged 18 years or older in which surgery for RRD was performed. Exclusion criteria included previous retinal detachment, tractional or exudative detachment, eye trauma, and any other ocular or retinal comorbidities. Patients who did not give informed consent were also excluded. All the surgeries were carried out by a single experienced retinal surgeon (SDN).

### 2.2. Data Collection

At baseline (preoperative), all the participants underwent BCVA assessment using Snellen charts, with data converted to the logMAR scale. When a patient could not read the letters from a distance of 1 m, BCVA was assessed by counting fingers or detecting hand movements and was converted to logMAR values of 1.8 or 2.4, respectively. Additional evaluations included slit-lamp biomicroscopy and dilated fundoscopy. The parameters recorded encompassed demographic details (age, gender, urban or rural residence), and symptom duration measured from onset to surgery (0–3 days, 4–7 days, 8–14 days and more than 15 days). The status of the crystalline lens (phakic, pseudophakic), macular condition (on or off), presence of PVR, epiretinal membrane, and macular hole were documented. Specific preoperative features of RRD such as location and extent of detachment, retinal tears, location and number were also noted. Comorbidities like diabetes mellitus and hypertension were recorded. PVR staging was made according to the updated classification of the Retina Society Terminology Committee (1991) [19].

### 2.3. Surgical Procedure

The surgical procedure was performed under local anesthesia. A 3-port PPV was conducted in all patients, using 23-gauge instrumentation and a non-contact wide-angle viewing system. After the removal of the central and peripheral vitreous, a 360° scleral indentation was performed to shave the vitreous base up to the ora serrata, followed by the removal of all vitreous traction from retinal tears. A complete fluid–air exchange was executed, simultaneously with subretinal fluid aspiration. Retinal breaks were treated by cryopexy. At the end of the surgery, silicone oil (1000 Centistokes) endotamponade was used. The target period for removing the silicone oil tamponade was approximately three months. Combined cataract surgery and vitrectomy were performed if visualization of the retina was inadequate due to lens opacity. In 21 patients, cataract surgery with intraocular lens implantation was performed during follow-up. The surgery was considered successful only if the retina stayed fully attached at six months of follow-up after a single procedure.

### 2.4. Statistical Analysis

Statistical analysis was done using R version 4.5.1 (R Core Team, Viena, Austria) [20] with the following packages: stringr version 1.6.0 [21], ggplot2 version 4.0.2 [ [22], MASS version 7.3-65 [23], openxlsx version 4.2.8.1 [24]. Numeric data was presented as mean ± standard deviation, and as median (1st quartile–3rd quartile) respectively. Categorical data was presented as absolute and relative frequencies, rounded to the first decimal.

The univariate LogMAR BCVA analysis included comparisons between groups at each measurement time (admission, one week after the intervention, one month after the intervention, three months after the intervention and six months after the intervention) based on the Wilcoxon rank sum test and Cliff’s delta as effect size, as well as comparisons from the baseline to subsequent post-intervention measurements within groups based on the Wilcoxon signed rank sum and Cliff’s delta effect size. LogMAR BCVA values were graphically presented using boxplots, where the horizontal lines represent the mean, the lower and upper sides of the rectangle represent the 1st and 3rd quartiles respectively, the whiskers represent the minimum or maximum values (excluding outliers), the dots represent the outliers (further than 1.5 times inter-quartile range from their respective 1st or 3rd quartiles), the diamond represents the mean and the inward triangles represent the standard deviation relative to the mean.

As the linear regression assumptions were not met (mainly due to non-normal residuals and heteroscedasticity in the LogMAR BCVA values across demographics and clinical categories), we used the robust fitting algorithm for linear models available through the rlm function in the MASS package in R.

We studied three sets of predictors: the first set (entitled pre-admission) contains demographics (biological gender male or female, age measured in years, and place of residence urban or rural) and medical history (time from symptom onset to intervention measured in days, diabetes present or absent, and hypertension present or absent). The second set (entitled post-evaluation) contains post-admission parameters (LogMAR BCVA at admission numeric, macula on or off, pseudophakia present or absent, PVR present (A or B) or absent, number of retinal tears, lattice degeneration present or absent, number of detached quadrants). PVR A (minimal) was defined clinically by slight vitreous haze and pigment clumps (“tobacco dust”), and PVR B (moderate) by wrinkling of the retinal surface, rolled edges of the retinal tear, folds on the retinal surface, retinal stiffness and retinal vessel tortuosity [25]. No PVR grade C was identified in our series. The third set (entitled full) contains all predictors from the previously mentioned models, as well as whether the patients had undergone phacoemulsification during the 6-month follow-up.

Each predictor was incorporated into a univariate model, using the 6-month LogMAR BCVA value as the predicted outcome. Then, because we found that macula-on and macula-off patients exhibit different progression patterns, we developed bivariate models that included each predictor, macula status (on/off), and the interaction between predictor and macula status. Finally, we created three multivariate models, each representing one of the predictor sets. These models did not include interaction terms, as this could have led to models with more predictors than observed values.

For the univariate LogMAR BCVA analysis across groups and measurement times, the results were considered statistically significant at *p* < 0.05. In the robust linear regression models, we reported the coefficients, 95% confidence intervals, and *p*-values for each predictor.

## 3. Results

### 3.1. Baseline Characteristics

A retrospective analysis was conducted on a consecutive series of 135 patients (136 eyes) fulfilling the predefined inclusion criteria, who underwent surgical intervention at our institution between January 2021 and December 2024. One male patient presented with bilateral rhegmatogenous retinal detachment; accordingly, each eye was analyzed as an independent unit. Laterality distribution revealed right eye involvement in 76 cases (55.9%) and left eye involvement in the remaining 60 cases (44.1%). The mean age was 64.551 ± 11.034 years, with a median (first quartile, third quartile) of 65 (59.75–72), ranging from 23 to 89 years old. Gender distribution comprised 74 female eyes (54.4%) and 62 male eyes (45.6%), with a slight predominance of female patients (54.41%) across the cohort. With respect to geographic origin, 87 eyes (64.0%) belonged to patients residing in urban areas, while 49 eyes (36.0%) were from rural regions (Table 1).

Descriptive statistics for the pre- and intra-operative factors examined are shown in Table 2.

Regarding lens status, 100 eyes (73.53%) were phakic at the time of presentation. Macula was off in 111 eyes (81.62%). The duration of symptoms from onset to surgery was highly variable, averaging 12.88 days ± 14.05 SD (range from 2 to 94 days). Preoperative BCVA was 1.52 logMar (±0.76 SD) and postoperative BCVA at 6 months after surgery was 0.74 logMar (±0.37 SD). The primary anatomical success rate was achieved in 123 patients, while 13 patients required additional surgery due to retinal redetachment. After the second surgery, all patients achieved final retinal reattachment at 6 months of follow-up.

### 3.2. Univariate Analysis of LogMAR BCVA Values During the Follow-Up Period

#### 3.2.1. Gender

**Figure 1:**
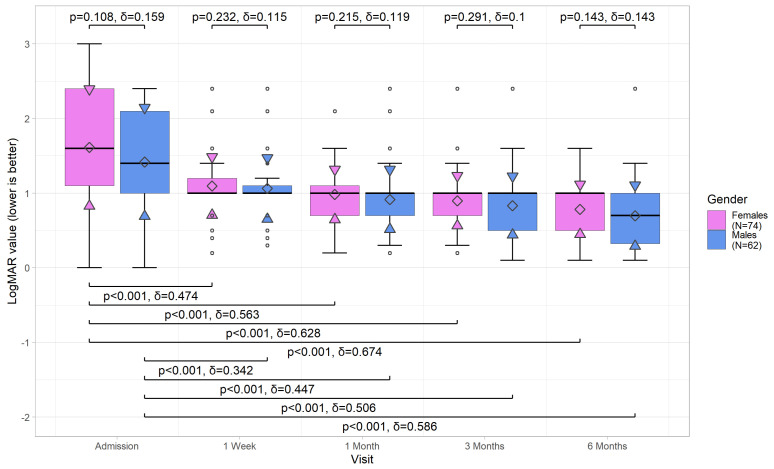
A boxplot of LogMAR BCVA values, by patient sex. The diamonds represent the mean, the inward triangles represent the standard deviation relative to the mean, and the values which are further than 1.5 times the inter-quartile range from the 1st or 3rd quartiles are considered outliers and are represented with dots. The upper side of the graph shows between-group comparisons at each measurement time using Wilcoxon rank sum and Cliff’s Delta (δ). The lower side of the graph shows within-group comparisons between the baseline and subsequent measurements using Wilcoxon signed rank and Cliff’s Delta (δ).

LogMAR BCVA values for the female gender at admission were 1.612 ± 0.785, with a median of 1.6 (1.1–2.4), while the mean for the male gender was 1.418 ± 0.729, with a median of 1.4 (1–2.1). At 6 months, females had a mean of 0.782 ± 0.336 with a median of 1 (0.5–1), and males had a mean of 0.698 ± 0.41 with a median of 0.7 (0.325–1). Although males in the current study appeared to present with and maintain lower LogMAR BCVA values during follow-up, there were no statistically significant differences between genders at any measured time. Both females and males showed statistically significant decreases from the baseline to later measurements, with females experiencing slightly larger decreases (Cliff’s Delta effect size of δ = 0.474 at one week after intervention and δ = 0.674 at 6 months) than males (δ = 0.342 at one week after intervention and δ = 0.586 at 6 months) (Figure 1).

#### 3.2.2. Age

**Figure 2:**
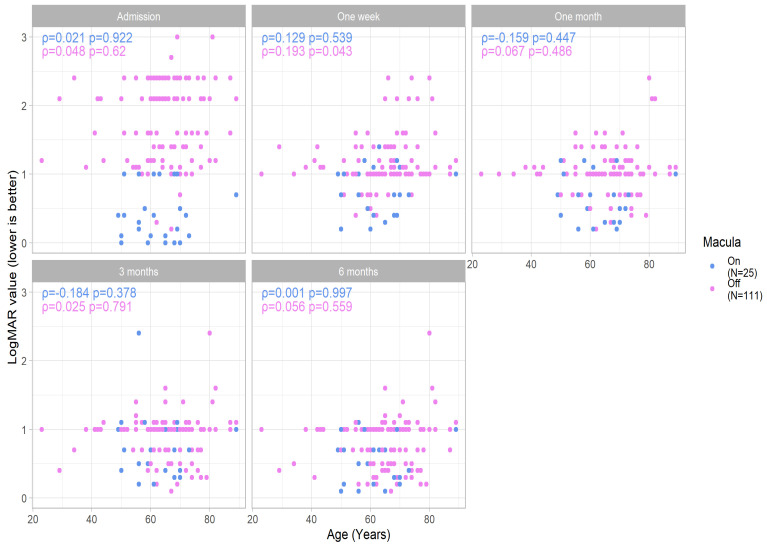
A scatterplot between age and LogMAR BCVA values, by measurement time and Macula on/off status, with Spearman correlation (ρ) and its associated statistical test (*p*-value) between age and the LogMAR values at each timepoint and for each group.

The average age of the subjects was 64.551 ± 11.034 years, with a median of 65 (59.75–72) years. The patients with macula on were slightly younger (mean 62.36 ± 9.358, median 61 (56–69)) than those with macula off (mean 65.045 ± 11.357, median 66 (60–72)), Wilcoxon rank sum *p* = 0.075. Except a weak, marginally significant correlation at one week after intervention in the macula-off group, there were no statistically significant correlations between age and LogMAR BCVA values (Figure 2).

#### 3.2.3. Residence

**Figure 3:**
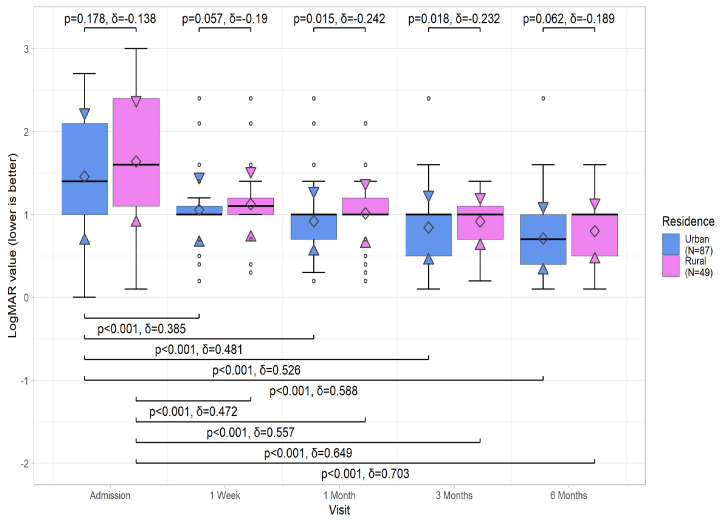
A boxplot of LogMAR BCVA values, by patient residence. The diamonds represent the mean, the inward triangles represent the standard deviation relative to the mean, and the values which are further than 1.5 times the inter-quartile range from the 1st or 3rd quartiles are considered outliers and are represented with dots. The upper side of the graph shows between-group comparisons at each measurement time using Wilcoxon rank sum and Cliff’s Delta (δ). The lower side of the graph shows within-group comparisons between the baseline and subsequent measurements using Wilcoxon signed rank and Cliff’s Delta (δ).

Regarding residence, the patients from urban areas had a mean LogMAR BCVA of 1.457 ± 0.773, a median of 1.4 (range 1–2.1), and at 6 months post-intervention, a mean of 0.713 ± 0.387 with a median of 0.7 (0.4–1). The patients from rural areas had a mean of 1.641 ± 0.74, a median of 1.6 (1.1–2.4), and, 6 months after intervention, a mean of 0.8 ± 0.342 with a median of 1 (0.5–1). Both groups showed statistically significant improvement, with the rural group having a larger effect size (δ = 0.703) than the urban group (δ = 0.588), but there was no statistically significant difference in the absolute LogMAR decrease between groups (Wilcoxon rank sum test *p* = 0.527, δ = −0.066) (Figure 3).

#### 3.2.4. Durations of Symptoms

**Figure 4:**
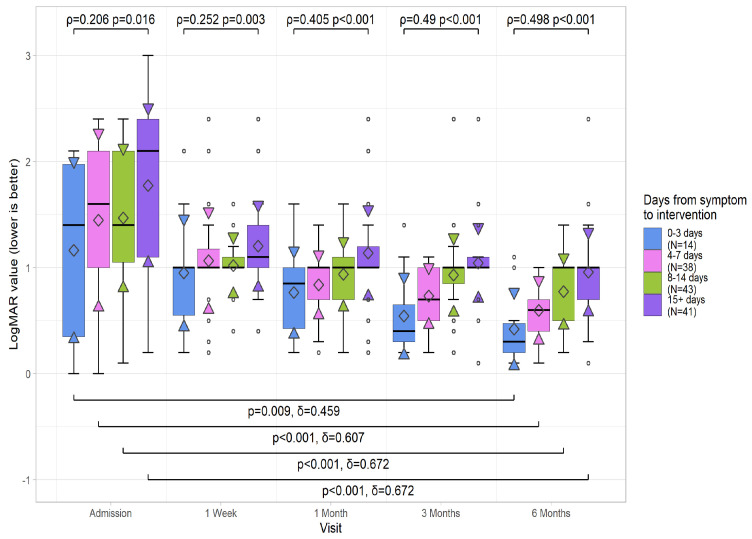
A boxplot of LogMAR BCVA values, by days from symptom onset to intervention. The diamonds represent the mean, the inward triangles represent the standard deviation relative to the mean, and the values which are further than 1.5 times the inter-quartile range from the 1st or 3rd quartiles are considered outliers and are represented with dots. The upper side of the graph shows Spearman correlation (ρ) and its associated statistical test (*p*-value) between the number of days and the LogMAR values at each timepoint. The lower side of the graph shows within-group comparisons between the baseline LogMAR values and measurements at 6 months after intervention using Wilcoxon signed rank test and Cliff’s Delta (δ).

Regarding the number of days elapsed from symptom onset until intervention, there was a correlation between the number of days and LogMAR BCVA (ρ = 0.206, *p* = 0.016), with a stronger correlation at 6 months after the intervention (ρ = 0.498, *p* < 0.001). Both the patients who presented early and those who presented late saw significant improvements in vision at 6 months, with those with a later admission and intervention having a slightly bigger benefit (*p* < 0.001, δ = 0.672) than those presenting early (*p* = 0.009, δ = 0.459) (Figure 4).

#### 3.2.5. Macular Status

**Figure 5:**
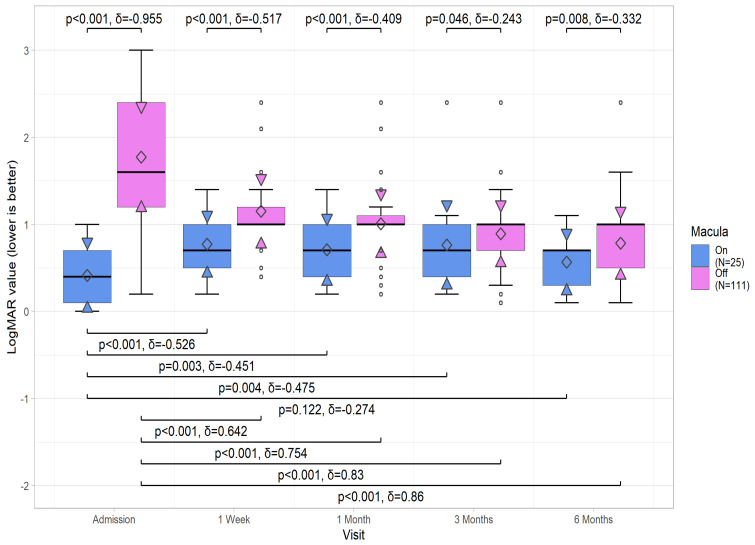
A boxplot of LogMAR BCVA values, by macula status. The diamonds represent the mean, the inward triangles represent the standard deviation relative to the mean, and the values which are further than 1.5 times the inter-quartile range from the 1st or 3rd quartiles are considered outliers and are represented with dots. The upper side of the graph shows between-group comparisons at each measurement time using Wilcoxon rank sum and Cliff’s Delta (δ). The lower side of the graph shows within-group comparisons between the baseline and subsequent measurements using Wilcoxon signed rank and Cliff’s Delta (δ).

Regarding macula status, the patients presenting with macula on had a mean LogMAR BCVA of 0.416 ± 0.383 and a median of 0.4 (0.1–0.7), while those with macula off had a mean of 1.773 ± 0.585 and a median of 1.6 (1.2–2.4). There was a statistically significant, strong difference between the groups (Wilcoxon rank sum *p* < 0.001, δ = −0.955). After the intervention, the patients with macula on experienced a significant decrease in vision quality (Wilcoxon signed-rank *p* < 0.001, δ = −0.526), with a one-week mean LogMAR BCVA of 0.772 ± 0.335 and a median of 0.7 (0.5–1). Over time, these patients regained vision, with a six-month mean of 0.568 ± 0.333 and a median of 0.7 (0.3–0.7), which was not significantly different from baseline (Wilcoxon signed-rank *p* = 0.122, δ = −0.274). The patients with macula off showed immediate benefit after intervention, with a one-week mean LogMAR BCVA of 1.15 ± 0.38 and a median of 1 (1–1.2), representing a moderate, significant improvement from baseline (Wilcoxon signed-rank *p* < 0.001, δ = 0.642). At six months post-intervention, this group had a mean of 0.784 ± 0.371 and a median of 1 (0.5–1), indicating substantial recovery compared to the baseline (Wilcoxon signed-rank *p* < 0.001, δ = 0.86). Throughout all measurement points, the patients with macula on at presentation maintained better vision than those with macula off (Figure 5).

#### 3.2.6. PVR

**Figure 6:**
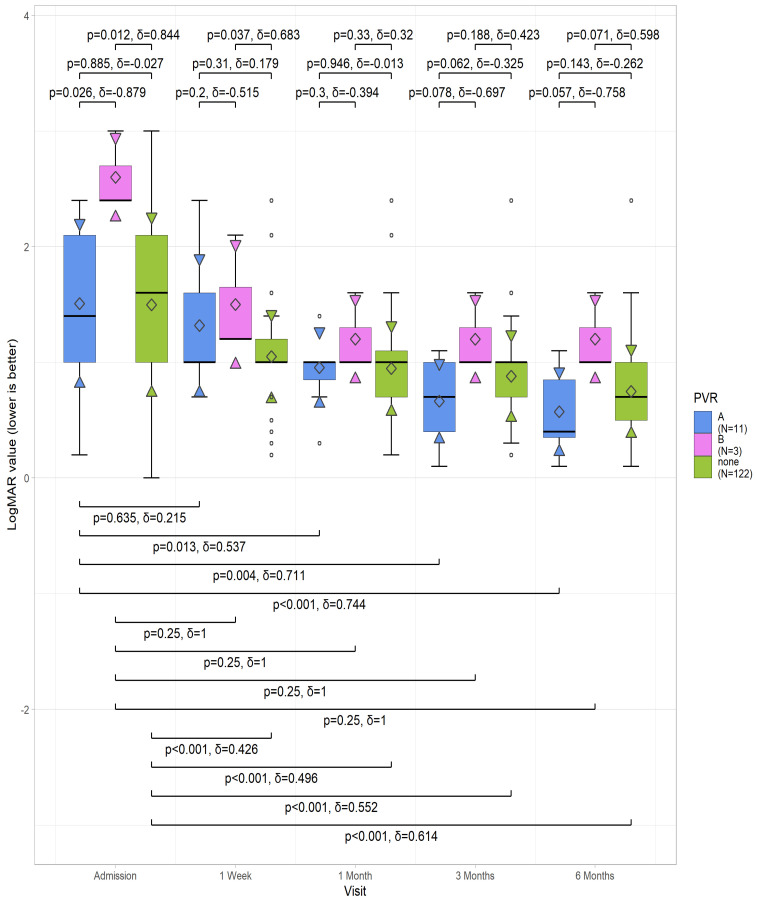
A boxplot of LogMAR BCVA values, by PVR. The diamonds represent the mean, the inward triangles represent the standard deviation relative to the mean, and the values which are further than 1.5 times the inter-quartile range from the 1st or 3rd quartiles are considered outliers and are represented with dots. The upper side of the graph shows between-group comparisons at each measurement time using Wilcoxon rank sum and Cliff’s Delta (δ). The lower side of the graph shows within-group comparisons between the baseline and subsequent measurements using Wilcoxon signed rank and Cliff’s Delta (δ).

Regarding PVR status, the three patients with PVR B had the worst vision at admission (mean LogMAR of 2.6 ± 0.346, median 2.4 (2.4–2.7)) compared to both PVR A (mean 1.509 ± 0.693, median 1.4 (1–2.1); *p* = 0.026, δ = −0.879) and no PVR (mean 1.498 ± 0.761, median 1.6 (1–2.1); *p* = 0.012, δ = 0.844). All the groups showed improvement, although the PVR B group maintained slightly higher values than the others (0.05 < *p* < 0.1 in Wilcoxon rank sum tests for LogMAR BCVA at 6 months), and its improvement could not be statistically confirmed due to the small sample size (Figure 6).

#### 3.2.7. Number of Tears

**Figure 7:**
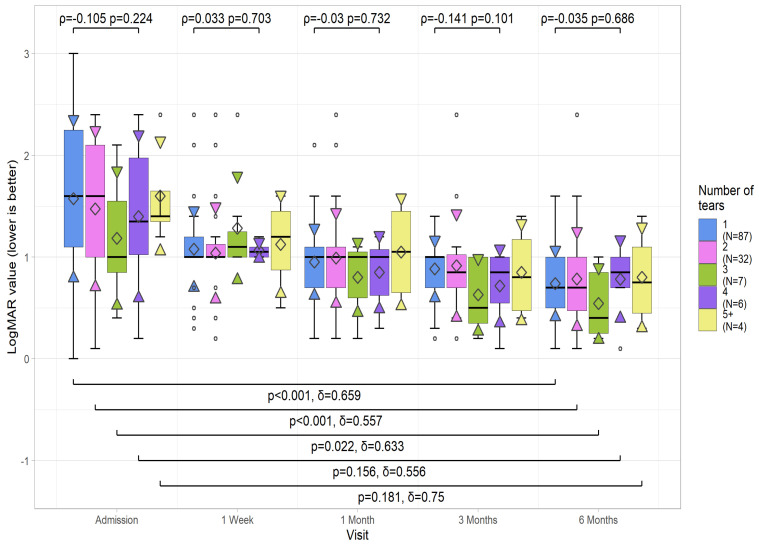
A boxplot of LogMAR BCVA values, by number of retinal ruptures. The diamonds represent the mean, the inward triangles represent the standard deviation relative to the mean, and the values which are further than 1.5 times the inter-quartile range from the 1st or 3rd quartiles are considered outliers and are represented with dots. The upper side of the graph shows Spearman correlation (ρ) and its associated statistical test (*p*-value) between the number of ruptures and the LogMAR values at each timepoint. The lower side of the graph shows within-group comparisons between the baseline LogMAR values and measurements at 6 months after intervention using Wilcoxon signed rank test and Cliff’s Delta (δ).

The number of retinal tears was not significantly correlated with LogMAR BCVA values at any measurement time point. Regardless of the number of retinal tears, all the groups showed improvements in vision (δ = 0.556–0.75), although the groups with a higher number of breaks did not reach statistical significance due to the small number of subjects in those groups (Figure 7).

#### 3.2.8. Detachment Extension

**Figure 8:**
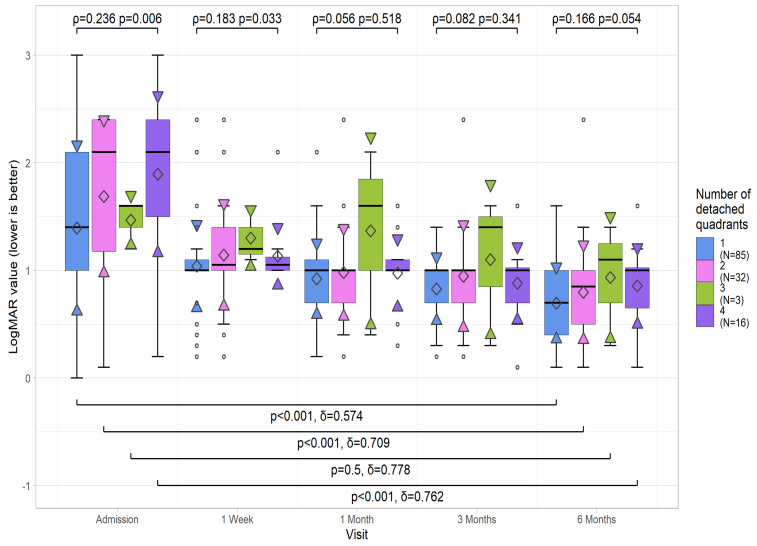
A boxplot of LogMAR BCVA values, by number of detached quadrants. The diamonds represent the mean, the inward triangles represent the standard deviation relative to the mean, and the values which are further than 1.5 times the inter-quartile range from the 1st or 3rd quartiles are considered outliers and are represented with dots. The upper side of the graph shows Spearman correlation (ρ) and its associated statistical test (*p*-value) between the number of detached quadrants and the LogMAR values at each timepoint. The lower side of the graph shows within-group comparisons between the baseline LogMAR values and measurements at 6 months after intervention using Wilcoxon signed rank test and Cliff’s Delta (δ).

The number of detached retinal quadrants had a weak correlation with LogMAR values at admission (ρ = 0.236, *p* = 0.006), which decreased and became non-significant at 6 months after intervention (ρ = 0.166, *p* = 0.054). All the groups (except the few patients with three detached quadrants *N* = 3) observed statistically significant improvements in vision at 6 months compared to the baseline, which were stronger in the groups with a higher number of detached quadrants (four detached quadrants: *p* < 0.001, δ = 0.762) compared to a lower number of detached quadrants (one detached quadrant: *p* < 0.001, δ = 0.574) (Figure 8).

#### 3.2.9. Lattice Degeneration

**Figure 9:**
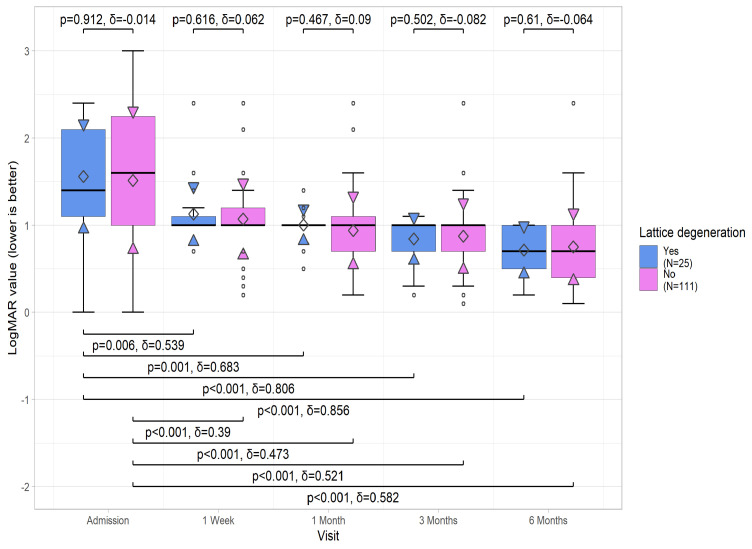
A boxplot of LogMAR BCVA values, by lattice degeneration. The diamonds represent the mean, the inward triangles represent the standard deviation relative to the mean, and the values which are further than 1.5 times the inter-quartile range from the 1st or 3rd quartiles are considered outliers and are represented with dots. The upper side of the graph shows between-group comparisons at each measurement time using Wilcoxon rank sum and Cliff’s Delta (δ). The lower side of the graph shows within-group comparisons between the baseline and subsequent measurements using Wilcoxon signed rank and Cliff’s Delta (δ).

The patients with lattice degeneration (baseline mean 1.56 ± 0.606 and median 1.4 (1.1–2.1)) had comparable results (*p* = 0.912, δ = −0.014) with those without lattice degeneration (baseline mean 1.515 ± 0.797 and median 1.6 (1–2.25)), and no statistically significant differences arose during the follow-up, such that both groups had statistically significant better vision at 6 months following surgery (*p* < 0.001) (Figure 9).

**Figure 10:**
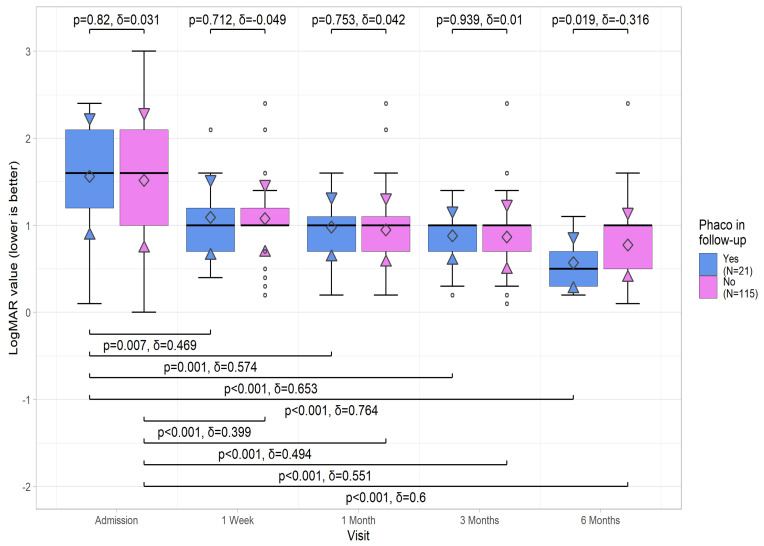
A boxplot of LogMAR BCVA values for the patients who had or did not have phaco during follow-up. The diamonds indicate the mean, the inward triangles show the standard deviation relative to the mean, and the outlier values more than 1.5 times the inter-quartile range from the first or third quartiles are shown as dots. The upper part of the graph displays between-group comparisons at each measurement time using Wilcoxon rank sum and Cliff’s Delta (δ). The lower part shows within-group comparisons between the baseline and subsequent measurements using Wilcoxon signed-rank and Cliff’s Delta (δ).

During the follow-up, some patients required phacoemulsification due to cataract development. Although there were no statistically significant differences in the LogMAR BCVA values at admission (*p* = 0.82), at 6 months, the patients who had phacoemulsification during the follow-up period showed significantly lower LogMAR BCVA values (mean 0.571 ± 0.302, median 0.5 (0.3–0.7)) than those who did not have it (mean 0.776 ± 0.377, median 1 (0.5–1). Both groups experienced vision improvements compared to baseline (*p* < 0.001) (Figure 10).

**Figure 11:**
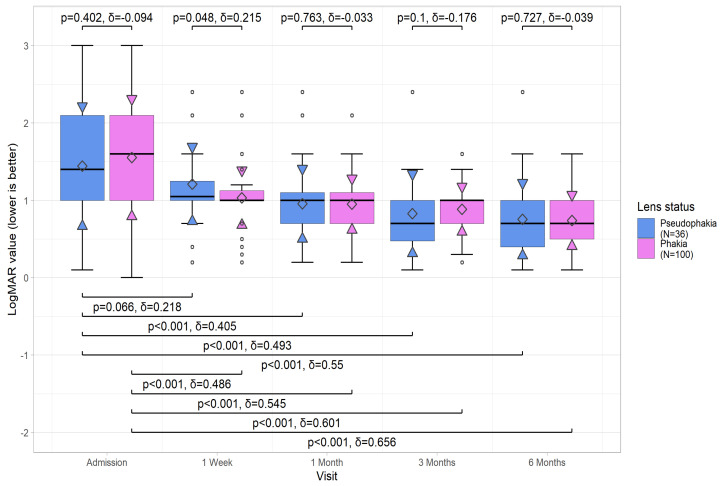
A boxplot of LogMAR BCVA values, by lens status. The diamonds represent the mean, the inward triangles represent the standard deviation relative to the mean, and the values which are further than 1.5 times the inter-quartile range from the 1st or 3rd quartiles are considered outliers and are represented with dots. The upper side of the graph shows between-group comparisons at each measurement time using Wilcoxon rank sum and Cliff’s Delta (δ). The lower side of the graph shows within-group comparisons between the baseline and subsequent measurements using Wilcoxon signed rank and Cliff’s Delta (δ).

Regarding lens status, 100 (73.5%) subjects had natural lens (phakia), while 36 (26.5%) had pseudophakia at admission (Figure 11). Except a marginal statistically significant difference at one week after admission (*p* = 0.048, δ = 0.215, small effect size in favor of the phakic group), there were no statistically significant differences between the groups, and both groups had significant improvements in their vision. In the pseudophakic group, macula was off in 30 eyes with a mean BCVA of 1.633 logMAR (±0.680 SD) at presentation, and a mean BCVA of 0.776 logMAR (±0.478 SD) at 6 months follow-up. Macula was on in six eyes, with a mean BCVA of 0.483 logMAR (±0.302) at presentation, and a mean BCVA of 0.7 logMAR (±0.378 SD) at 6 months follow-up. The lens status was not significantly correlated with LogMAR BCVA at 6 months.

The demographic and retinal detachment characteristics of pseudophakic eyes are presented in Table 3.

### 3.3. Medical History

**Figure 12:**
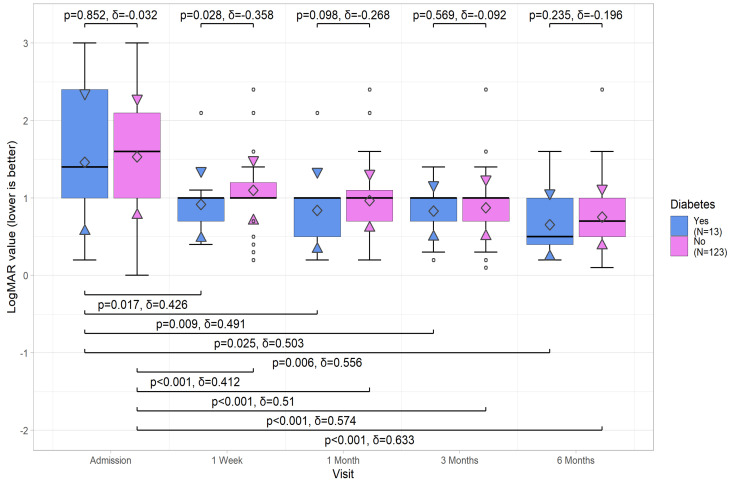
A boxplot of LogMAR BCVA values, by diabetes mellitus history. The diamonds represent the mean, the inward triangles represent the standard deviation relative to the mean, and the values which are further than 1.5 times the inter-quartile range from the 1st or 3rd quartiles are considered outliers and are represented with dots. The upper side of the graph shows between-group comparisons at each measurement time using Wilcoxon rank sum and Cliff’s Delta (δ). The lower side of the graph shows within-group comparisons between the baseline and subsequent measurements using Wilcoxon signed rank and Cliff’s Delta (δ).

There were no statistically significant differences between the groups regarding diabetes mellitus history, and both groups had their vision improved 6 months after the intervention (Figure 12).

**Figure 13:**
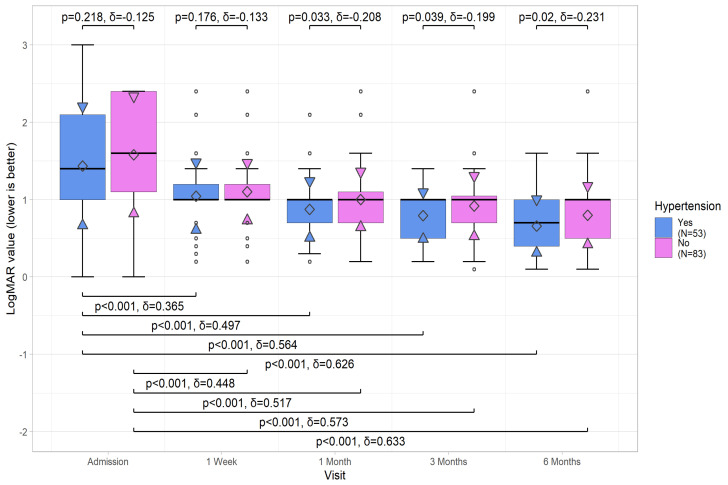
A boxplot of LogMAR BCVA values, by hypertension history. The diamonds represent the mean, the inward triangles represent the standard deviation relative to the mean, and the values which are further than 1.5 times the inter-quartile range from the 1st or 3rd quartiles are considered outliers and are represented with dots. The upper side of the graph shows between-group comparisons at each measurement time using Wilcoxon rank sum and Cliff’s Delta (δ). The lower side of the graph shows within-group comparisons between the baseline and subsequent measurements using Wilcoxon signed rank and Cliff’s Delta (δ).

The patients with systemic hypertension had slightly lower (*p* = 0.218, δ = −0.125) LogMAR BCVA values at admission (mean 1.436 ± 0.77, median 1.4 [1–2.1]) compared to those without it (mean 1.58 ± 0.759, median 1.6 [1.1–2.4]). Although both groups experienced statistically significant improvements (*p* < 0.001), there was a small (δ = −0.231), yet significant, difference in LogMAR values at 6 months, with hypertensive patients (mean 0.658 ± 0.348, median 0.7 [0.4–1]) exhibiting lower scores than non-hypertensive patients (mean 0.799 ± 0.38, median 1 [0.5–1]); see Figure 12. However, when adjusting for baseline, there were no statistically significant differences between the groups at 6 months (*p* = 0.709, δ = −0.038) (Figure 13).

### 3.4. Robust Linear Regression Models

We analyzed the effect of pre-admission patient characteristics on LogMAR BCVA values at 6 months using robust linear regression models. When analyzing univariate models, the time elapsed from symptom onset to surgical intervention was statistically significant (*p* < 0.001), with each day adding, on average, 0.01 units to the LogMAR BCVA value at 6 months after surgery; the presence of systemic hypertension was also a significant predictor (*p* = 0.036), patients with hypertension having, on average, 6-month LogMAR BCVA values lower by 0.135 compared to those without hypertension. The patients from urban areas also had, on average, lower values by 0.104 compared to those from rural areas, this predictor having a tendency towards statistical significance (*p* = 0.098). Because we observed different patterns of recovery between the patients with macula on and those with macula off, we also analyzed bivariate models based on each predictor, the macula on/off status, and the predictor–macular status interaction, with both time from symptom onset to surgery as well as systemic hypertension being significant predictors for 6-month LogMAR BCVA values (*p* < 0.001 and *p* = 0.04, respectively). Lastly, we created a multivariate model including all pre-admission patient characteristics as predictors, with time elapsed from symptom onset to surgery (*p* < 0.001, coefficient = 0.01) and systemic hypertension (*p* = 0.033, coefficient = −0.137) being statistically significant (Table 4).

The second set of predictors analyzed consisted of post-admission ophthalmological characteristics. In univariate models, the admission LogMAR BCVA values had significant predictive power (*p* < 0.001, coefficient = 0.195), meaning that, on average, for each point increase in LogMAR BCVA at admission, the LogMAR BCVA at 6 months would be higher by 0.195. This relationship was also present in the multivariate model. Macular status was a significant predictor in univariate models (*p* = 0.01, coefficient = −0.206), indicating that, on average, the patients with the macula on had a 6-month LogMAR BCVA value 0.206 lower than those with the macula off. However, this relationship was not significant in the multivariate model (*p* = 0.278). Lastly, the number of detached quadrants was a significant predictor of 6-month LogMAR BCVA values (*p* = 0.046, coefficient = 0.061), but this relationship was not maintained in the multivariate model that accounts for all post-admission ophthalmological characteristics (Table 5).

Lastly, we analyzed a multivariate model including all previously used characteristics and whether the patients had phacoemulsification during the 6-month follow-up as predictors. Of those, only the time elapsed from symptom onset to surgery (*p* < 0.001, coefficient = 0.007), LogMAR BCVA value at admission (*p* < 0.001, coefficient = 0.172), and phaco during follow-up were significant. Systemic hypertension was not statistically significant (*p* = 0.092, coefficient = −0.102) (Table 6).

## 4. Discussion

The prognosis for visual and anatomical outcomes in RRD depends on various factors that can influence results. Modern surgical techniques, such as PPV, reduce surgical trauma, decrease postoperative inflammation, and shorten recovery times, resulting in very high reattachment success rates. Many prognostic factors have been examined in previous studies. Reports have indicated that postoperative BCVA is linked to preoperative macular status [16,26,27], preoperative BCVA [9,26,28,29], duration of symptoms [18,30,31], extent of retinal detachment [18,32], macular hole [12,17], and PVR [12,18,32,33].

In our retrospective study, we analyzed 136 eyes from 135 patients who underwent surgery for RRD. Our statistical analysis suggests that BCVA at presentation, duration of symptoms, and phacoemulsification during follow-up are statistically correlated with postoperative BCVA at 6 months. Initial BCVA was significantly better in macula-on versus macula-off cases and univariate analysis showed a strong difference between the macula-on and macula-off groups. Nevertheless, macular status did not remain an independent significant factor in the final multivariate model, after adjusting for potential confounders such as duration of RRD and baseline BCVA. The explanation of this paradoxical finding may be found in the misclassification of macular status which was done retrospectively according exclusively to clinical criteria which can be inaccurate. “Macula-on” may include subclinical/shallow foveal involvement that can only be demonstrated with optical coherence tomography (OCT). In addition, very recent cases (˂24–48 h) were part of the “macula-off” group. Consequently, there could have been an overlap between groups, resulting in the loss of statistical significance. These confounding factors originate from the retrospective nature of the study and do not entitle us to state that macular status does not affect the postoperative outcome. Our robust multivariate linear regression analysis demonstrated that the patients with better BCVA at presentation achieved better functional outcomes after surgery, meaning that, on average, for each point in LogMAR BCVA at admission, at 6 months the patient would have a LogMAR BCVA value higher by 0.172. Poulsen et al. [29] demonstrated that a poorer BCVA at presentation (logMAR > 0.3) is a significant predictor of poor final vision outcome. Benda et al. [28] found that preoperative BCVA was the most statistically significant prognostic factor; on average, a point increase in admission BCVA determined an increase of 0.369 in LogMAR BCVA at 6 months.

The crucial preoperative symptom duration was 7 days in the study by Chatziralli et al. [18], where a duration longer than 1 week was associated with worse postoperative visual outcomes. Conversely, in the study by Baudin et al. [34], patients operated on in less than 12 days had better BCVA (on average 75 ETDRS letters, corresponding to a logMAR of 0.2) compared to those operated on after more than 12 days (on average 71 ETDRS letters, corresponding to a logMAR of 0.3). Hirata et al. [35] found no significant correlation between symptom duration and postoperative visual outcome. Our report indicates a significant correlation between symptom duration and postoperative BCVA, with each additional day associated with a 0.01-unit increase in logMAR BCVA at 6 months after surgery. The patients who presented within the first 0–3 days after the symptoms’ onset had better BCVA at 6 months than the ones who presented after more than 15 days.

The preoperative factors that showed no significant association with functional outcome in our study were age, gender, residence, lens status and macular status (on/off). Due to the distribution of PVR in which 122 eyes had no PVR, 11 eyes had grade A, and three grade B; we were unable to obtain a sufficiently strong statistical significance to demonstrate that PVR was a negative prognostic factor, as has already been demonstrated in the literature. For instance, Chatziralli et al. [18] reported that PVR was a significant prognostic factor for poor postoperative BCVA, similarly to Park et al. [32] (*p* < 0.001) and Zgolli et al. [33] (*p* < 0.005). Chatziralli et al. [18] found that increasing age was significantly associated with worse postoperative BCVA. Regarding the lens status, we found no statistical correlation with visual outcomes, but we observed a positive statistical correlation between phacoemulsification during follow-up and postoperative BCVA. In medical history, we found no correlation between diabetes mellitus and postoperative BCVA at 6 months, but we observed a tendency towards statistical significance of the association between arterial hypertension and postoperative BCVA. The patients with hypertension had, on average, 6-month LogMAR BCVA values that were lower by 0.102 compared to those without hypertension. These associations may be explained by ACE inhibitors (primarily treatment for arterial hypertension in our patients), which provide significant retinal protection by inhibiting the local renin–angiotensin system. Further studies should aim to take into consideration the medication of the patients before symptom onset, in order to provide a better context of the patient’s disease.

In our study, univariate analysis of the macular status showed a different pattern of recovery between patients with macula on and those with macula off. Therefore, we believe that regression-based analyses should consider this characteristic. Still, in the final multivariate model, we did not find a statistical relationship between macular status at presentation and postoperative BCVA at 6 months. However, Altindal et al. [26] and Barequet et al. [27] found that preoperative and postoperative BCVA were significantly better in eyes with macula-on retinal detachment. Unlike our findings, which showed no statistical link between the extent of retinal detachment or the number, position, and dimensions of retinal tears and postoperative BCVA, Guner et al. [31] demonstrated that patients with four quadrants of retinal detachment had significantly lower BCVA at 12 months follow-up compared to those with one quadrant of detachment. Sung et al. [17] reported that preoperative BCVA (logMAR) was 2.23 (±0.45) for patients with total RRD and 0.82 (±0.83) for partial RRD, and final BCVA (logMAR) was 1.88 (±0.83) for total RRD and 0.35 (±0.52) for partial RRD, with a success rate of 75% for total RRD and 96.6% for partial RRD.

Our study has certain limitations, such as its retrospective design. We did not routinely perform optical coherence tomography before surgery, and we do not have data about OCT biomarkers. Additional prospective research involving a larger patient cohort, more detailed retinal imaging and better capturing of the patients’ history and medication is necessary to better identify prognostic factors for patients undergoing surgery for RRD.

## 5. Conclusions

The present study adds to the growing body of evidence identifying symptom duration and preoperative BCVA as important prognostic factors for visual recovery following PPV in RRD. Lens and macular status, extent of retinal detachment and number of tears did not influence the postoperative outcome in our series.

The originality in our study does not come from the topic itself (which is well studied), but from demonstrating with the help of multivariate logistic regression models that functional outcome depends on combined structural and temporal factors rather than individual predictors. Our paradoxical finding that macular status did not significantly influence BCVA should be interpreted cautiously. An original result that deserves further investigation, although it did not reach statistical significance, is represented by the better functional outcomes in patients with hypertension.

The main clinical message of our research is that surgical timing and duration of RRD may be more important than macular status alone when predicting visual outcomes, highlighting the importance of early diagnosis and surgery to optimize visual recovery.

## Figures and Tables

**Table 1:** Eye characteristics.

Gender (*n*)	Age (y)	Residence	Macular Status	Preop. BCVA	Postop. BCVA
Female (74)	65.24 ± 9.73 (range 29–87)	U:47 R:27	On:13 Off:61	1.612 ± 0.785 SD	0.782 ± 0.336 SD
Male (62)	63.73 ± 12.44 (range 23–89)	U:40 R:22	On:12 Off:50	1.418 ± 0.729 SD	0.698 ± 0.41 SD

BCVA = Best corrected visual acuity. R = Rural. U = Urban.

**Table 2:** Clinical characteristics of patients included in the study.

Factor Studied	Frequency (%)
Lens status	
Phakic	100 (73.53%)
Pseudophakic	36 (26.47%)
Duration of symptoms from onset to surgery	
0–3 days	14 (10.29%)
4–7 days	38 (27.94%)
8–14 days	43 (31.62%)
15 days or more	41 (30.15%)
Macular status	
On	25 (18.38%)
Off	111(81.62%)
PVR	
PVR A	11 (8.09%)
PVR B	3 (2.21%)
NO PVR	122 (89.70%)
Extent of detachment	
1 quadrant	85 (62.50%)
2 quadrants	32 (23.53%)
3 quadrants	3 (2.21%)
4 quadrants	16 (11.76%)
Location of retinal detachment	
Superior	36 (26.47%)
Inferior	22 (16.17%)
Nasal	14 (10.30%)
Temporal	8 (5.88%)
Total	13 (9.55%)
Inferior, nasal, temporal	6 (4.44%)
Superior, nasal, temporal	3 (2.20%)
Superior, temporal, inferior	4 (2.94%)
Superior, temporal	27 (19.85%)
Superior, nazal	3 (2.20%)
Number of tears	
1	87 (63.97%)
2	32 (23.53%)
3	7 (5.15%)
4	6 (4.41%)
More than 4	4 (2.94%)
Tears dimensions	
Small	141
Medium	63
Large	11
Giant	2
Location of tears	
Superior	110
Temporal	42
Nasal	14
Inferior	27
More than 1 quadrant involved	24
Lattice degeneration	
Yes	25 (18.38%)
No	111 (81.62%)
Epiretinal membrane	
Yes	4 (2.94%)
No	132 (97.06%)
Macular hole	
Yes	1 (0.74%)
No	135 (99.26%)
HTN	
Yes	82 (60.30%)
No	53 (39.70%)
Diabetes mellitus	
Yes	13 (9.56%)
No	122 (90.44%)

PVR = Proliferative vitreoretinopathy. HTN = Hypertension.

**Table 3:** Demographics and retinal detachment characteristics of pseudophakic eyes.

Factor Studied	Frequency (%)
Gender	
F	17 (47.22%)
M	19 (52.78%)
Residency	
U	23 (63.88%)
R	13 (36.12%)
Days of symptoms from onset to surgery	
0–3	3 (8.33%)
4–7	12 (33.33%)
8–14	9 (25.01%)
More than 15 days	12 (33.33%)
Macular status	
On	6 (16.66%)
Off	30 (83.34%)
PVR	
PVR A	6 (16.66%)
None	30 (83.34%)
Lattice degeneration	
Yes	7 (19.44%)
No	29 (80.56%)
Number of tears	
1	18 (50%)
2	11 (30.55%)
3	3 (8.33%)
4	3 (8.33%)
5	1 (2.79%)
Extent of detachment	
1 quadrant	20 (55.55%)
2 quadrants	12 (33.33%)
4 quadrants	4 (11.12%)

**Table 4:** Robust linear regression models for LogMAR BCVA values after 6 months.

	Univariate Model		Bivariate Model						Multivariate Model	
	The Predictor		The Predictor		Macula (on Compared to off)		Predictor-Macula Interaction		The Predictor	
	Coefficient (95% CI)	*p*-Value	Coefficient (95% CI)	*p*-Value	Coefficient (95% CI)	*p*-Value	Coefficient (95% CI)	*p*-Value	Coefficient (95% CI)	*p*-Value
Sex (females compared to males)	0.1 (−0.025–0.224)	0.118	0.102 (−0.03–0.234)	0.129	−0.181 (−0.404–0.042)	0.111	−0.036 (−0.343–0.272)	0.819	0.072 (−0.041–0.186)	0.211
Age (years)	0.002 (−0.004–0.008)	0.477	0.001 (−0.005–0.007)	0.665	−0.307 (−1.376–0.762)	0.571	0.002 (−0.015–0.019)	0.843	0.004 (−0.001–0.01)	0.105
Provenience (urban compared to rural)	−0.104 (−0.227–0.019)	0.098	−0.091 (−0.231–0.049)	0.202	−0.208 (−0.521–0.106)	0.193	0.026 (−0.339–0.391)	0.889	−0.068 (−0.185–0.05)	0.258
Time elapsed from symptom onset to surgery	0.01 (0.006–0.014)	<0.001	0.009 (0.005–0.013)	<0.001	−0.271 (−0.496–0.047)	0.018	0.013 (−0.007–0.032)	0.198	0.01 (0.005–0.014)	<0.001
Diabetes	−0.129 (−0.34–0.082)	0.228	−0.188 (−0.445–0.07)	0.152	−0.241 (−0.428–0.053)	0.012	0.228 (−0.423–0.879)	0.49	−0.039 (−0.245–0.166)	0.706
Hypertension	−0.135 (−0.26–−0.009)	0.036	−0.142 (−0.277–0.007)	0.04	−0.233 (−0.44–−0.026)	0.028	0.091 (−0.215–0.397)	0.557	−0.137 (−0.263–0.011)	0.033

Univariate models use each predictor individually to predict the 6-month value. Bivariate models use the specified predictors (one by one) together with the macula on/off status and the predictor–macular status interaction term to predict the 6-month value. The multivariate model uses all predictors specified, without interactions.

**Table 5:** Robust linear regression models for LogMAR BCVA values after 6 months.

	Univariate Model		Bivariate Model						Multivariate Model	
	The Predictor		The Predictor		Macula (on Compared to off)		Predictor–Macula Interaction		The Predictor	
	Coefficient (95% CI)	*p*-Value	Coefficient (95% CI)	*p*-Value	Coefficient (95% CI)	*p*-Value	Coefficient (95% CI)	*p*-Value	Coefficient (95% CI)	*p*-Value
LogMAR BCVA at admission	0.195 (0.121–0.27)	<0.001	0.231 (0.125–0.338)	<0.001	0.081 (−0.197–0.36)	0.564	0.041 (−0.323–0.406)	0.822	0.235 (0.126–0.343)	<0.001
Macula (on compared to off)	−0.206 (−0.361–−0.051)	0.01							0.12 (−0.098–0.337)	0.278
Pseudophakia	−0.025 (−0.168–0.117)	0.726	−0.071 (−0.221–0.079)	0.351	−0.261 (−0.44–0.082)	0.005	0.252 (−0.11–0.613)	0.171	0.02 (−0.115–0.155)	0.767
PVR (A/B compared to none)	−0.055 (−0.267–0.157)	0.61	−0.099 (−0.311–0.113)	0.358	−0.218 (−0.382–−0.054)	0.009	0.236 (−0.528–1)	0.542	−0.118 (−0.317–0.082)	0.244
No. of ruptures	−0.008 (−0.072–0.055)	0.799	−0.018 (−0.08–0.045)	0.579	−0.309 (−0.681–0.063)	0.102	0.072 (−0.16–0.304)	0.54	0.005 (−0.055–0.064)	0.873
Lattice degeneration	−0.021 (−0.186–0.145)	0.807	−0.068 (−0.235 -0.1)	0.424	−0.229 (−0.396–−0.061)	0.008	0.211 (−0.341–0.764)	0.451	0.014 (−0.141–0.169)	0.859
No. of detached quadrants	0.061 (0.001–0.121)	0.046	0.042 (−0.023–0.106)	0.207	−0.169 (−0.772–0.434)	0.58	−0.007 (−0.539–0.526)	0.98	0.028 (−0.035–0.091)	0.388

Univariate models use each predictor individually to predict the 6-month value. Bivariate models use the specified predictors (one by one) together with the macula on/off status and the predictor–macular status interaction term to predict the 6-month value (for the macula status predictor, no bivariate model was analyzed, as this would cause the predictor itself to be doubled). The multivariate model uses all predictors specified, without interactions.

**Table 6:** Multivariate robust linear regression model for LogMAR BCVA values after 6 months.

Predictor	Coefficient (95% CI)	*p*-Value
Sex (females compared to males)	0.064 (−0.044–0.171)	0.242
Age (years)	0.003 (−0.002–0.008)	0.235
Provenience (urban compared to rural)	−0.073 (−0.186–0.04)	0.205
Time elapsed from symptom onset to surgery	0.007 (0.003–0.011)	<0.001
Diabetes	−0.031 (−0.224–0.163)	0.754
Hypertension	−0.102 (−0.221–0.017)	0.092
LogMAR BCVA at admission	0.172 (0.071–0.273)	<0.001
Macula (on compared to off)	0.08 (−0.118–0.278)	0.424
Pseudophakia	−0.024 (−0.149–0.101)	0.706
PVR (A/B compared to none)	−0.132 (−0.314–0.05)	0.154
No. of ruptures	−0.007 (−0.062–0.047)	0.789
Lattice degeneration	0.006 (−0.136–0.148)	0.936
No. of detached quadrants	0.013 (−0.044–0.071)	0.644
Phaco during follow-up	−0.194 (−0.35–0.038)	0.016
Intercept (free term)	0.257 (−0.146–0.659)	0.209

The model includes only the mentioned predictors, with no interaction term.

## Data Availability

The original contributions presented in this study are included in the article. Further inquiries can be directed to the corresponding author.

## Abbreviations

The following abbreviations are used in this manuscript:

BCVA	Best corrected visual acuity
SB	Scleral buckle
PPV	Pars plana vitrectomy
RPE	Retinal pigment epithelium
PVR	Proliferative vitreoretinopathy
RRD	Rhegmatogenous retinal detachment
